# A Review of Motion and Orientation Processing in Migraine

**DOI:** 10.3390/vision3020012

**Published:** 2019-03-27

**Authors:** Alex J. Shepherd

**Affiliations:** Department of Psychological Sciences, Birkbeck, University of London, Malet St, London WC1E 7HX, UK; a.shepherd@bbk.ac.uk; Tel.: +44-2076316212

**Keywords:** migraine, motion processing, orientation processing

## Abstract

Visual tests can be used as noninvasive tools to test models of the pathophysiology underlying neurological conditions, such as migraine. They may also be used to track changes in performance that vary with the migraine cycle or can track the efficacy of prophylactic treatments. This article reviews the literature on performance differences on two visual tasks, global motion discrimination and orientation, which, of the many visual tasks that have been used to compare differences between migraine and control groups, have yielded the most consistent patterns of group differences. The implications for understanding the underlying pathophysiology in migraine are discussed, but the main focus is on bringing together disparate areas of research and suggesting those that can reveal practical uses of visual tests to treat and manage migraine.

## Introduction

Current estimates are that one in five women and one in eleven men have migraine [1]. The World Health Organisation lists migraine as the 20th most disabling lifetime condition (9th for women), based on the number of years lost to disability [2]. Another report cites headache disorders as the 9th most disabling condition generally, and the 5th for women [3]. In the UK, it is estimated that 90,000 people cannot attend work or education each day because of headache, amounting to 25 million workdays or schooldays lost every year. The Migraine Trust [4] and the Migraine Action Association (MAA) [5] estimate the cost as £2.25 billion a year. Furthermore, over 50% of people surveyed by the MAA felt that migraine had adversely affected their careers, and one third kept the fact they had migraine secret, fearing discrimination at work. Evidently, the cost of migraine on personal, occupational, educational and social interactions is high. Understanding differences between migraine and control groups may help in the management of migraine so that the impact on personal, occupational, educational and social interactions can be minimised (for a commentary, see reference [6]).

Visual processing in migraine has been examined due to: (i) the intense sensitivity to light that patients experience (photophobia) [7]; (ii) the visual disturbances that may precede an attack (visual aura) [7]; (iii) the fact that visual stimuli (such as high contrast stripes, other high contrast regular geometric patterns, flicker or glare), can trigger attacks in up to 60% of patients [6,8]; (iv) the discomfort that can be experienced from these potential visual triggers, in between attacks [6,8,9] and (v) correlations between visual discomfort, endorsement of visual triggers and performance on certain visual tasks [8,9,10,11,12,13,14,15]. Moreover, there is some evidence that performance on various visual tasks varies with migraine duration (years experienced), attack proximity, attack frequency [10,16,17,18,19] and treatment efficacy [20].

Visual psychophysical tests can be used as non-invasive tools to test models of the pathophysiology underlying neurological conditions such as migraine. A difficulty with psychophysical studies, however, is selecting tasks and displays where performance can be unambiguously attributed to processing at particular stages or within particular pathways in the visual system or deciphering what the cause or causes of demonstrable differences may be [14,21]. Nevertheless, research has shown differences between migraine and control groups, between attacks, using diverse visual tasks, which have been attributed to anomalous processing at various stages within the visual system. Some studies have highlighted anomalies within retinal or subcortical retinofugal pathways [22,23], however, much of the research has concentrated on tasks that putatively show anomalous processing within the primary visual (striate) cortex and, subsequently, on tasks that could be attributed to processing in higher (extrastriate) visual cortical areas (reviewed in reference [21]). 

Of the many visual tests that have been used to understand cortical processing differences between migraine and control groups, the perception of motion and orientation has yielded the most consistent patterns of group differences, although inconsistencies do exist in the literature [10,11,12,13,15,21,24,25,26,27,28,29,30,31,32]. These studies will be illustrated below. Several studies have reported impaired global or coherent motion discrimination in migraine [11,24,25,26,27,28,32]. These studies have used displays in which a cloud of signal dots drift in a defined direction and are interspersed with, over sequential trials, a growing proportion of other, noise, dots, moving in random directions, until a threshold for reliable discrimination of coherent motion from the signal dots is reached. 

**Figure 1:**
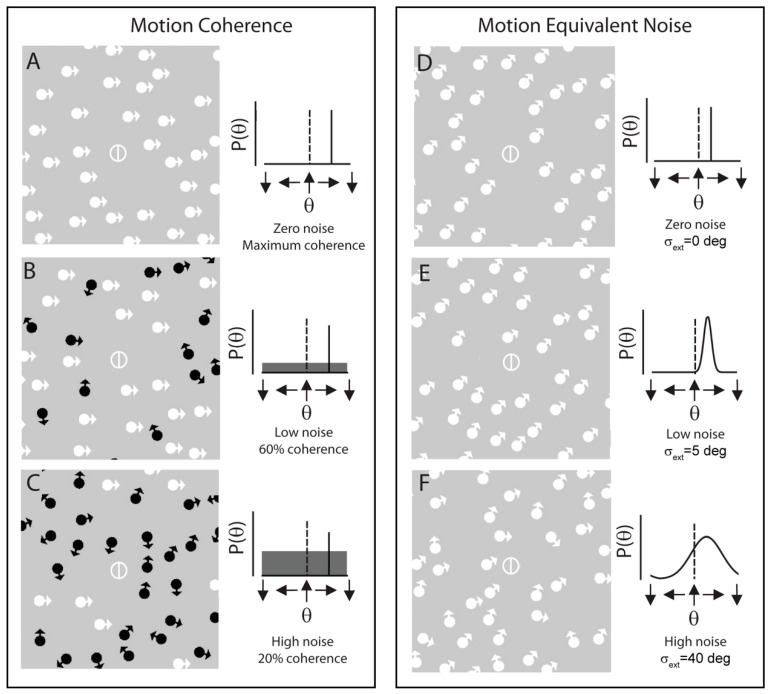
An example of the displays used for motion discrimination tasks, using motion coherence (**A**–**C**) and equivalent noise (**D**–**F**) paradigms. In both tasks, the participant must classify the signal direction (or average signal direction) as right or left of vertical. In the motion coherence paradigm, external noise was added to the stimulus by assigning a subset of dot random directions. In the equivalent noise paradigm, external noise was added to the stimulus by increasing the standard deviation of motion directions displayed by the noise dots. In A–C, directions of signal motion are indicated by white arrows; directions of noise motion are indicated by black arrows. (In the actual experiment all signal and noise elements were comprised of white dots. Noise dots are shown in black for illustration purposes only.) Associated signal and noise probability distribution functions are presented alongside each example display; in these, the reference direction is denoted by a dotted black line and the signal probability density function is denoted by a solid black line. Adapted from Tibber et al. [32].

While coherent motion discrimination, with signal nots embedded in random noise, has been the traditional approach to assess global motion perception in migraine, an alternative approach is to ask what sort of noise is relevant to add to a display. Motion coherence has been typically assessed with noise dots moving in random directions. Equivalent noise paradigms, instead, use interspersed noise dots that are associated with the direction of motion of the signal dots [for an example see Figure 1, adapted from reference [32], which shows typical motion coherence displays (A–C) and, for comparison, motion equivalent noise displays (D–F)]. Motion coherence is impaired in migraine, but the equivalent noise paradigm, which yields two estimates of neural noise: (i) internal (baseline, no external noise and (ii) sampling adequacy (the ability to pool global motion signals) have been reported to be equivalent in migraine and control groups. Nevertheless, motion coherence thresholds were found to be impaired in migraine [32] leading to the conclusion that there is an inability to exclude irrelevant noise in migraine.

A third example is to introduce noise that has a temporal element but no coherent motion signal, such as adding dynamic random noise (“twinkle”) to a coherent motion display. In a relative motion task, dynamic noise aided performance, in migraine only, lowering thresholds for motion direction discrimination [12]. Evidently, the type of noise added to discrimation tasks needs further research and clarification as to what relevant noise is in the visual system and whether it is differentially affected in clinical conditions, as the cited literature suggests.

**Figure 2:**
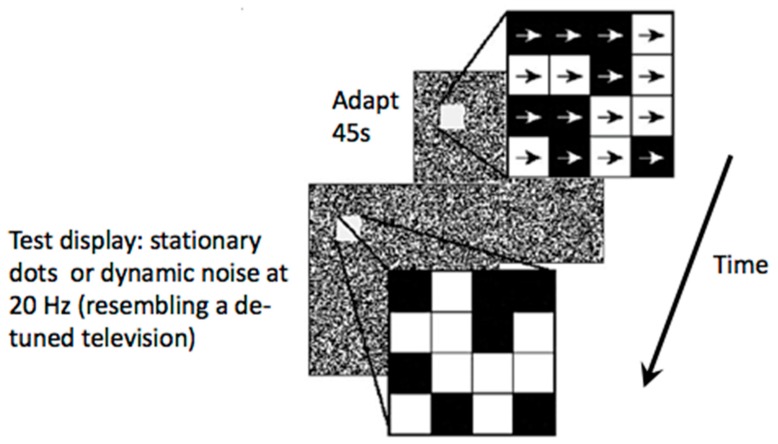
An example of the displays used to assess prolonged motion after-effects in migraine. Adapted from Shepherd [10,11].

In addition to impaired global/coherent motion discrimination, extended motion after-effects (MAE) have also been reported for both local and global motion percepts [10,11,13,14] (for an example, see Figure 2). These studies used random dot displays for adaptation and test conditions. In the adaptation condition, the dots drifted in one direction for 45 s. The test displays could either be a display of stationary random dots, or dynamically “twinkling” dots, which resembled a de-tuned television. A local MAE is seen with the stationary test display and a global MAE is seen with the dynamic, twinkling, test display. The MAE seen with stationary test displays appears to slide, like a lava flow, and does not look like real motion (one sees the motion yet knows the dots are stationary). The MAE seen with dynamic displays, on the other hand, looks like real motion, like billowing clouds. The MAE duration is extended with both types of displays in migraine.

**Figure 3:**
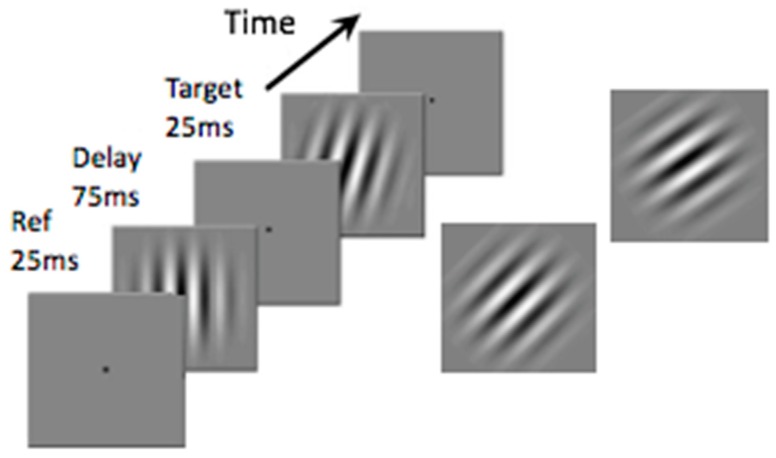
An example of the displays used to assess the oblique effect using gratings. In one condition the reference grating (“Ref”) is vertical and the test grating is oriented slightly clockwise or counter-clockwise to vertical. Thresholds for reliable discrimination can be determined. In a second condition, the reference is oriented at 45 degrees from vertical and orientation thresholds are again determined. Adapted from Tibber et al. [24].

**Figure 4:**
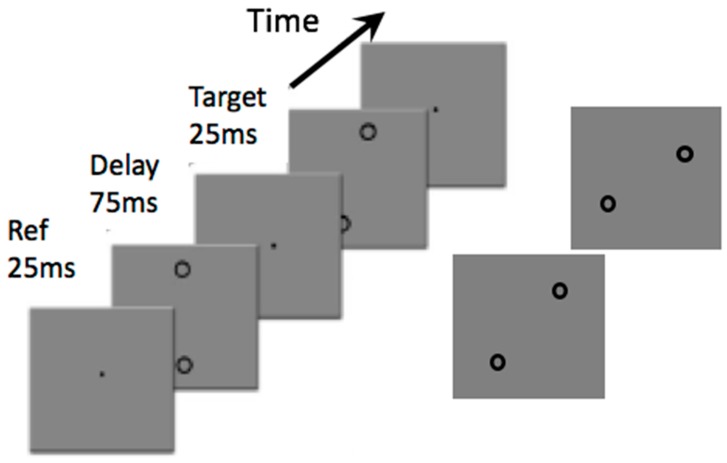
An example of the displays used to assess the oblique effect using virtual lines. As in Figure 3, in one condition the reference grating (“Ref”) is vertical and the test grating is oriented slightly clockwise or counter-clockwise to vertical. Thresholds for reliable discrimination can be determined. As in Figure 3, in the second condition, the reference is oriented at 45 degrees from vertical and orientation thresholds are again determined. Adapted from Tibber et al. [24].

Impaired detection and discrimination for certain orientation tasks have also been reported [24]. A classic test uses the oblique effect, usually assessed with lines or gratings, as shown in Figure 3. Discrimination of the differences in orientation of gratings oriented near vertical is usually very good: the procedure is to present a vertical grating as a reference and subsequently present another tilted slightly in a clockwise or counter-clockwise direction and determine the threshold for discriminating an orientation difference. With these displays there are no differences between migraine and control groups, discrimination thresholds are at around one degree clockwise or counter-clockwise from vertical. If the reference grating is presented at 45 degrees from vertical, however, and the task is then to judge whether the test grating is oriented clockwise or counter-clockwise from the reference, thresholds increase dramatically to around five degrees in the migraine group and just over three degrees in the control group. That study showed an enhanced oblique effect in migraine, with gratings. An interesting extension occurs using virtual lines (Figure 4). A virtual line can be imagined between two widely spaced circles. This condition was included to move performance differences beyond the primary visual cortex (V1) as no neurons in V1 have receptive fields large enough to respond to the two anchor widely spaced circles, yet a virtual line can be perceived or imagined. Exactly the same pattern of results occurred with virtual lines as occurred with the gratings: discrimination of orientation around vertical was just under two degrees for both groups. Discrimination for oblique virtual lines, however, increased to around six degrees for the migraine group and four degrees for the control group.

The results from both of these lines of research (global motion and virtual orientation judgments) have been attributed to differences in extrastriate cortical excitability in migraine [11,24]. 

Contradictory reports, showing no group differences, may be attributable to researchers who have not adequately considered other factors that might affect performance on global motion or orientation tasks. For example, several studies have used vertical gratings for both motion and orientation judgements which, it has been argued, anchors performance to processing in the primary visual cortex [11,13,21]. For example, one group [33] has reported no differences between migraine and control groups on a version of the MAE using drifting gratings as the adapt and test stimuli, thus confounding motion and orientation and likely anchoring performance to processing in the primary visual cortex. It was to avoid this confound that the earlier cited studies used random dot displays for adapting and test stimuli [10,11,13,14]. As a second example, input to striate or extrastriate cortical areas relies on the adequate processing of information in precortical pathways. Consequently, differences in early visual processing may have consequences that could be misattributed to differences in cortical processing. Apparently impaired global motion discrimination could reflect dysfunction that occurs earlier in the visual pathways than the cortex (reviewed in reference [12]).

For example, tests of contrast sensitivity, visual acuity, stereo acuity, visual discomfort, visual stress and visual triggers of migraine can be formally correlated with performance on motion or orientation tasks. Each of these measures taps into different levels of processing within the visual pathways, from the eye, to precortical and cortical visual areas, to cognitive stages. Singh and Shepherd [14] included an assessment of contrast sensitivity using horizontal gratings at four cycles per degree, close to the maximum sensitivity of the human visual system, in a relative motion, global motion detection, and global motion discrimination study. They reported their migraine group had significantly poorer contrast sensitivity, that is, they had higher contrast thresholds, than the control group. Contrast sensitivity also correlated significantly with performance on each motion task. As expected from previous work, these correlations showed that poorer contrast sensitivity was associated with fewer correct responses on a motion direction detection task and poorer performance (higher thresholds) on the global and relative motion discrimination tasks. When contrast sensitivity was added as a covariate to the analyses, the group differences for the motion direction detection thresholds and relative motion tasks disappeared. For their motion discrimination task, the group differences persisted, however, so it was concluded that there are cortical variations in migraine, in addition to impaired contrast sensitivity, and that anomalous processing in low-level (precortical) pathways can confound interpretation of performance on other tasks if not taken into account. Tests of precortical visual processing should be included to preclude the possibility of failing to recognize performance differences for nominally cortical tasks that can be attributed to processing differences in the earlier visual pathways that feed into the cortex. 

The importance of taking into account explanations of deficits in migraine at all levels of the visual system, even as early as the retina, has been highlighted by various authors [12,13,14,23,27,28,34], but not by all. Without this, it is not possible to determine to what extent a deficit in global motion or orientation perception can really be attributed to an abnormality in extrastriate cortical areas that code for global motion or orientation, or to an abnormality earlier in the visual system. Performance on global motion and orientation tasks that can be attributed to extrastriate processing has yielded a more consistent pattern of group differences, which is generally an impairment in migraine [11,12,13,24,31]. The underlying neural mechanism or mechanisms that can explain these differences is still debated, such as cortical hyper- or hypo-excitability in particular cortical areas (reviewed in reference [29]). Equally, the role of external noise or internal neural noise in these impairments in migraine requires further study [12,32,35].

Complementing these psychophysical studies, there are a number of imaging reports of structural and functional changes in motion processing cortical areas (V5/MT and V3A) between attacks, such as increased cortical thickness and cortical surface area and increased fMRI blood oxygenation level dependent responses to visual motion stimulation [36,37,38]. Whether these changes reflect developmental differences or occur as a consequence of multiple attacks requires further research [36,39]. Transcranial magnetic stimulation (TMS) studies over the motion area V5/MT also indicate differences between people with and without migraine in extrastriate cortical areas, as lower intensity TMS pulses were needed to elicit phosphenes in migraine [40]. Both of these lines of research, together with some of the psychophysical research, indicate that motion and orientation processing in extrastriate cortex differs in migraine, a result that may help in understanding and managing the condition. 

This review has focused on differences in motion and orientation perception that have been reported between migraine and control groups. This line of research has been used to refine models of neurological function in migraine, such as the debate between hyper- vs. hypo-excitability models of neural function, but this debate is not covered in detail here (see reference [21]). Rather, this review is concerned with the practical consequences or applications of demonstrable differences in visual processing in migraine. Many of the differences cited are measured by determining thresholds: the minimum proportion of coherently moving dots needed to perceive the direction of coherent motion in a random dot display containing coherently and randomly moving dots, or the smallest orientation difference between two oriented patterns that can be reliably discriminated. The consequences of the cited differences, do not, in general, affect a person’s day to day life. It is possible, however, that performance on these tasks may vary with the migraine cycle as has been reported for certain electrophysiological measures, notably a habituation deficit in particular visual evoked and event-related potentials (reviewed in reference [41]). If so, appropriate tests of motion or orientation discrimination may provide the elusive biomarker for migraine. To conclude, if appropriate tasks can be selected, tests of motion and/or orientation may be useful not only to understand differences between migraine and control groups and to aid diagnosis, but also to manage the condition by showing performance differences that track the migraine cycle and/or predict treatment efficacy in clinical trials (see for an example reference [42]).
